# Honeysuckle (*Lonicera japonica* Thunb.) Extract as a Natural Preservative for Rabbit Meat: Inhibition of Lipid and Protein Oxidation and Maintenance of Myofibrillar Protein Functionality

**DOI:** 10.3390/foods15152766

**Published:** 2026-08-06

**Authors:** Xiaohua Huang, Wenjiao Chen, Zhengqiang Tong, Peiyin Tang, Siyi Yang, Xin Zhang, Feixia Duan, Jiamin Zhang

**Affiliations:** 1 Meat Processing Key Laboratory of Sichuan Province, College of Food and Biological Engineering, Chengdu University, Chengdu 610106, China19136160820@163.com (W.C.); syyang11@163.com (S.Y.); 19113322921@163.com (X.Z.); 2 Sichuan Provincial Engineering Research Center of Meat Quality Improvement and Safety Control Technology, Chengdu 610106, China; 3 Zigong Eighty Salt Gang Food Co., Ltd., Zigong 643000, China; 18909009066@189.cn; 4 Sichuan Aishi Rabbit Food Co., Ltd., Bazhong 636700, China; 18882001151@163.com; 5 Department of Food Engineering, College of Biomass Science and Engineering, Sichuan University, Chengdu 610065, China; duanfeixia@126.com

**Keywords:** honeysuckle extract, rabbit meat, myofibrillar protein, oxidation

## Abstract

This study investigated the effects of honeysuckle extract (HE) on the oxidative stability, storage quality, and myofibrillar protein functionality of raw rabbit meat during a 5-day storage period at 4 °C. Raw rabbit meat was treated with HE (1–7% *w*/*v*; HE1–HE7) or deionized water (control) and stored for 5 days. pH and color parameters were analyzed, lipid and protein oxidation profile of raw rabbit meat were investigated, and the physicochemical characteristics of rabbit myofibrillar protein (MP) were analyzed. Results showed that HE treatment significantly decreased the levels of TBARS, TVB-N and TVC compared to the control group. Further studies revealed that HE treatment significantly reduced the protein carbonyl level while increasing sulfhydryl content. In addition, HE treatment increased the WHC and solubility and reduced the surface hydrophobicity of rabbit MP during refrigerated storage. Taken together, HE serves as a potent natural preservative for raw rabbit meat, effectively suppressing oxidative and microbial deterioration to maintain storage quality and extend shelf life.

## 1. Introduction

Rabbit meat is a high-quality meat source and has garnered substantial attention for bodybuilders, high-level meat consumers [1] and other special populations (infant complementary feeding, elderly diets, and cardiovascular disease management, etc.) [2] due to its highly favorable nutritional profile, including high protein content, low cholesterol, low fat, and a rich proportion of polyunsaturated fatty acids (PUFAs) [3]. However, rabbit meat is particularly susceptible to biochemical deterioration during storage due to higher protein and PUFA content [4,5], which leads to many problems such as rancidity, nutrient loss, and decreased economic value of rabbit meat products.

However, the commercial demand for long-term cold storage unavoidably promotes lipid breakdown, resulting in a radical chain reaction that targets both lipid and protein fractions. The initial phase is dominated by lipid peroxidation, generating aggressive reactive oxygen species (ROS) and secondary aldehydes such as malondialdehyde (MDA). These electrophilic aldehydes do not remain confined to the lipid fraction. Instead, they readily diffuse and form adducts with biological macromolecules, such as proteins and nucleic acids, leading to protein cross-linking and fragmentation [6]. This intense lipid–protein co-oxidation physically unfolds the MP, forcing the exposure of buried non-polar residues. These changes ultimately led to a loss of essential functional properties, including protein solubility, surface hydrophobicity, water-holding capacity (WHC), and higher carbonyl content, and faster denaturation [7,8], which reduce consumer acceptance and shorten shelf life.

To mitigate oxidative, microbial spoilage and extend shelf life, the meat industry commonly includes vacuum packaging [9], modified atmosphere packaging (such as filling with N_2_ and CO_2_) [10], and synthetic antioxidants (e.g., butylated hydroxytoluene and butylated hydroxyanisole) [11]. However, consumers are increasingly concerned about the potential health risks associated with chemical additives. Therefore, the development of safe and efficient natural antioxidants has become an important direction in the field of meat products [12]. Recently, natural plant extracts, such as green tea [13], grape seed [14], and rosemary extracts, have been demonstrated to be effective in meat preservation due to their strong antioxidant capacity [15].

Plant extracts are obtained through targeted extraction technologies that capture specific active components from plants while maximally preserving their natural structures and functions, aligning with the current market demand for green and safe food additives. Honeysuckle (*Lonicera japonica* Thunb.), a well-known dietary and medicinal botanical resource in East Asia, is highly abundant in chemical components, including bioactive polyphenols, flavonoids and chlorogenic acids [16]. These active compounds have exhibited significant antibacterial, anti-inflammatory, and antioxidant activities [17]. Furthermore, our previous studies revealed that HE can effectively inhibit lipid oxidation in pre-treated rabbit meat [16]. However, the physicochemical characteristics affecting the interactions between HE and MP, as well as its application for improving the shelf-life and oxidative stability of raw rabbit meat, remains to be further studied.

Therefore, this study investigated the effects of HE on the oxidative stability, storage quality, and MP functionality of raw rabbit meat during a 5-day storage period at 4 °C. The study comprised three main components: (1) An investigation of the dose-dependent effects of HE on conventional freshness indicators (pH, color parameters) in raw rabbit meat. (2) An evaluation of the efficacy of HE on oxidative stability and preserving qualities in raw rabbit meat. (3) An analyzation of the physicochemical characteristics of rabbit MP. The study provides critical theoretical foundations and establishes a molecular basis for utilizing HE as a clean-label stabilizer in raw meat products.

## 2. Materials and Methods

### 2.1. Chemicals and Reagents

Chemicals including kalium chloratum (KCl), magnesium chloride (MgCl_2_) and sodium chloride (NaCl) were purchased from Tianjin Zhiyuan Chemical Reagent Co., Ltd. (Tianjin, China). Thiobarbituric acid (TBA), trichloroacetic acid (TCA), magnesium oxide and sodium dihydrogen phosphate (Na_2_HPO_4_) were purchased from Chron chemicals (Chengdu, China). Ethylene glycol tetraacetic acid (EGTA), hydrochloric acid (HCl) and ethanol solvent were purchased from Fuchen (Tianjin, China) Chemical Reagent Co., Ltd. (Tianjin, China), Chengdu Jinshan Chemical Reagent Co., Ltd. (Chengdu, China) and Tianjin Jindong Tianzheng Fine Chemical Reagent Factory (Tianjin, China), respectively. Bromophenol blue (BPB) was purchased from Shanghai Macklin Biochemical Co., Ltd. (Shanghai, China). All other chemicals and reagents were of analytical grade or higher.

### 2.2. Preparation of HE

Preparation and extraction procedures of the dried flowers of honeysuckle samples (Nanjiang Liangyuan Food Co., Ltd., Bazhong, China) were conducted following previously documented protocols [16]. Briefly, the dried flowers of honeysuckle samples were washed, air-dried, ground into fine powder, and passed through a 60-mesh sieve. Subsequently, 5 g of honeysuckle powder was placed in a round-bottom flask, followed by 65% ethanol solvent at a solid-to-liquid ratio of 1:25 (*w*/*v*). The extraction conditions were as follows: reflux at 65 °C and 60 rpm for 1 h, followed by filtration to collect the supernatant, and the residual powder was extracted twice more.

### 2.3. Preparation of Rabbit Meat

Rabbit meats were obtained from Ha Wu Ye Food Co., Ltd. (Leshan, China), which were cut into pieces (3 cm × 3 cm × 3 cm) after washing. Then, samples were treated with 1%, 2%, 3%, 4%, 5%, 6%, and 7% (*w*/*v*) HE (HE1, HE2, HE3, HE4, HE5, HE6, and HE7, respectively) for 1 h. Meanwhile, rabbit meat treated with deionized water represented the control samples. All samples were then drained out, put in separate plastic containers, and stored at 4 °C for 5 days. This 5-day period was selected since rabbit meat is highly susceptible to oxidation and typically spoils within 5–7 days under normal aerobic refrigeration.

### 2.4. Determination of the pH and Color

The pH values were measured according to GB 5009.237-2016 methods [18]. Samples were homogenized twice through a mincer with an aperture not exceeding 4 mm. A total of 5 g of the homogenate was transferred to a beaker, 0.1 mol/L KCl solution was added at a solid-to-liquid ratio of 1:10 (*w*/*v*), and this was homogenized at 12,000 r/min. Prior to measurement, the pH meter was calibrated with standard commercial phthalate buffer (pH 4.00) and phosphate buffer (pH 6.86). The combined electrode was immersed in the sample suspension and the reading was recorded to the nearest 0.01 unit once stabilized. Each sample was analyzed in triplicate; the arithmetic mean was calculated.

The color parameters including L* (brightness), a* (redness), and b* (yellowness) of rabbit meat were measured using a calibrated colorimeter (CR-400, Konica Minolta, Tokyo, Japan).

### 2.5. Thiobarbituric Acid Reactive Substance (TBARS) and Total Volatile Basic-Nitrogen (TVB-N) Assay

The TBARS and TVB-N contents were measured according to previously documented protocols. In summary, for TBARS, 5 g of rabbit meat was homogenized in 50 mL of 7.5% trichloroacetic acid (*w*/*v*) mixture using a shaker at 50 °C for 30 min. Subsequently, 5 mL of filtrate or standard was transferred to a new colorimetric tube and combined with 5 mL of 0.02 mol/L thiobarbituric acid solution. The mixture was then heated at 90 °C for 30 min, and the absorbance was measured at 532 nm with a spectrophotometer (UV-1100, Mapada Instruments, Shanghai, China). The dilution factor was multiplied by the known standards to calculate the TBARS value. For TVB-N, 20 g of the sample was homogenized using a homogenizer with 100 mL of distilled H_2_O. The mixture was impregnated, filtered, and 5 mL magnesium oxide was added. The sample was distilled for 5 min using a Kjeldahl Apparatus (KDN-102C, Shanghai xianjian instruments Co., Ltd., Shanghai, China), and then it was titrated with 0.01 mol/L HCl. The equation is as follows:

(1) $$TVB-N\;(mg/100\;g)=\frac{\left(V_{1}-V_{2}\right)\times c\times 14}{m\times \left(V∕V_{0}\right)}\times 100,$$

where V_1_ and V_2_ represent HCl volumes (mL) for the sample and blank respectively; V represents the volume of the aliquot filtrate taken for the actual distillation; V_0_ represents the total volume of the sample extract; and c and m represent the concentration of HCl standard titration solution and the mass of sample, respectively. Distilled H_2_O was used as a blank control in this experiment.

### 2.6. Microbiological Analysis

The total bacterial count (TVC) was determined by Lee et al., with slight modifications [19]. Briefly, 25 g of rabbit meat sample was homogenized in 225 mL of 0.85% sterile physiological saline with a homogenizer (HX-4M, Huxi, Shanghai, China). The mixture was then diluted and added into an agar plate counting medium and was incubated at 37 °C for 72 h.

### 2.7. Protein Carbonyl and Total Sulfhydryl Assay

Rabbit meat of protein carbonyl (BC1275) and total sulfhydryl (BC1375) contents were analyzed with commercial assay kits (Solarbio Science & Technology, Beijing, China) respectively, according to the manufacturer’s instructions. The absorbance was measured at 370 nm for protein carbonyl and 412 nm for total sulfhydryl on a plate reader (FlexA-200, Molecular Devices, Hangzhou, China).

### 2.8. Extraction of Rabbit Myofibrillar Proteins (MPs) and Preparation of Gels

Rabbit MP was extracted as described by Cao et al. with slight modifications [20]. For the extraction of rabbit MP, ground meat was homogenized in four volumes of ice-cold extraction buffer (0.1 mol/L Na_2_HPO_4_, 0.1 mol/L KCl, 2 mmol/L MgCl_2_, 1 mmol/L EGTA, pH 7.0). The protein lysate was centrifuged at 10,000× *g* at 4 °C for 15 min. This procedure was repeated three times. Then, the protein precipitation was homogenized in four volumes of ice-cold 0.1 mol/L NaCl solution, filtered, and centrifuged. The concentration of protein in the precipitation was measured using a BCA Protein Assay Kit (PC0020, Solarbio Science & Technology, Beijing, China). For preparation of rabbit MP gels, the extracted rabbit MP was diluted and mixed in 10 mL Eppendorf tubes with a solubilization buffer (25 mmol/L Na_2_HPO_4_, 0.6 mol/L NaCl, pH 6.25). The use of 0.6 mol/L NaCl provides the optimized high ionic strength strictly necessary to dissociate and thoroughly solubilize the MPs, which is a prerequisite for gelation. The mixture was then heated at 70 °C for 15 min. Subsequently, the samples were cooled to room temperature and stored at 4 °C overnight to obtain rabbit MP gels.

### 2.9. Protein Solubility Assay

The rabbit MP solution was diluted to 2 mg/mL protein solution with buffer solution and centrifuged at 5000× *g* for 15 min at 4 °C. The concentration of protein in the precipitation was measured using a BCA protein assay kit. The protein solubility was calculated using Equation (2).

(2) $$X(\%)=\frac{C_{1}}{C_{2}}\times 100\%,$$

where C_1_ and C_2_ represent the concentration of protein solution after centrifugation and concentration of protein solution before centrifugation (mg/mL), respectively.

### 2.10. Protein Hydrophobicity Assay

A total of 1.5 mL rabbit MP (5 mg/mL) solution was vortexed in 200 μL BPB buffer. The mixture was subsequently centrifuged at 4000× *g* for 10 min. The absorbance was measured at 595 nm on a plate reader (FlexA-200, Molecular Devices, Hangzhou, China). The protein hydrophobicity was calculated as:

(3) $$BPB\;(\mu g)=\frac{200\;\mu g\times \left(V_{0}-V\right)}{V}$$

where V_0_ and V represent the absorbance at 595 nm of the buffer and sample mixed with BPB solution after centrifugation, respectively.

### 2.11. Gel Properties

#### 2.11.1. Gel Strength Assay

The gel strength assay was performed according to the method described by Chelh et al. with slight modifications [21]. The rabbit MP gel strength was analyzed using a TA-XT Plus texture analyzer (Stable Micro Systems Ltd., Godalming, UK) equipped with a 0.5 cm probe (P/0.5) at room temperature. Samples were measured under the following conditions: pre-test speed = 5 mm/s, test speed = 1 mm/s, post-test speed = 5 mm/s, and trigger force = 5 g. The maximum peak force recorded exactly at the macroscopic fracture point of the gel was determined as the breaking force, which was reported as the gel strength.

#### 2.11.2. Gel Whiteness Assay

A chromameter (CR-400, Konica Minolta, Tokyo, Japan) was calibrated for sample determination. The L*, a*, and b* values of rabbit MP gel were recorded and figured using Equation (4).

(4) $$Whiteness=100-\sqrt{{\left(100-L^{*}\right)}^{2}}+a^{*2}+b^{*2}\;,$$

#### 2.11.3. WHC Assay

In brief, 5 g of rabbit MP gel was weighed and placed in 10 mL Eppendorf tubes, and then was centrifuged at 3000× *g* at 4 °C for 15 min. The WHC was calculated as follows:

(5) $$WHC\;(\%)=\frac{M_{2}}{M_{1}}\times 100\%,$$

where M_1_ and M_2_ are the rabbit MP gel weights (g) before and after centrifugation, respectively.

### 2.12. Statistical Analysis

Data were processed and analyzed by Excel and SPSS 26 software (Armonk, NY, USA). A two-way analysis of variance (ANOVA) procedure, followed by Tukey’s honestly significant difference (HSD) post hoc test, was performed to evaluate the main effects of the HE concentration, storage duration, and their interaction (HE concentration × storage time). All experiments were performed in triplicate, and the results are presented as the mean ± SE. *p*-values less than 0.05 were considered statistically significant.

## 3. Results

### 3.1. Effect of HE on the pH of Raw Rabbit Meat

**Figure 1:**
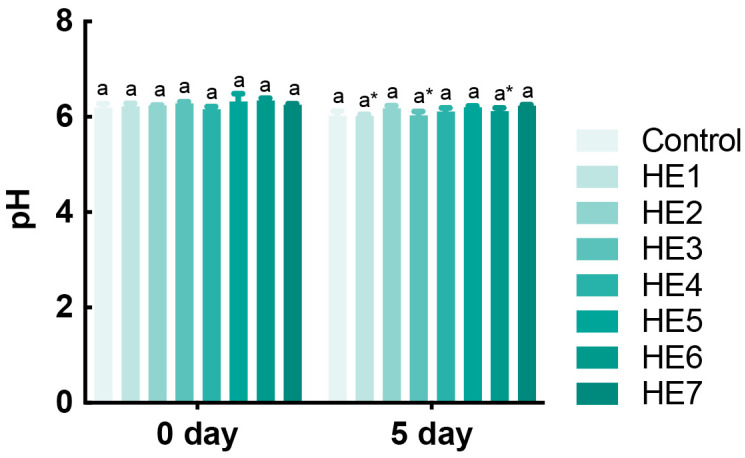
Change in the pH value of raw rabbit meat treated by different concentrations of HE treatment during refrigerated storage. Data are expressed as mean ± SE. Different lowercase letters indicate significant differences within the same storage time (*p* < 0.05) among groups. * Values within the same treatment indicate significant differences (*p* < 0.05).

The pH of rabbit meat is one of the conventional indicators used to reflect its freshness [22]. To determine the freshness of raw rabbit meat, the pH was determined. The raw rabbit meat was treated without and with HE treatment (HE1–HE7) at stored at 4 °C for days 0 and 5, as shown in Figure 1. The results showed that during days 0 and 5 of the refrigeration processes, HE treatment did not change the pH values of the raw rabbit meat compared with the control, with values ranging from 6.06 to 6.65 and 5.84 to 6.27 on days 0 and 5, respectively. In addition, the pH value of the raw rabbit meat was significantly decreased during the storage period only by HE1, HE3 and HE6 groups. These findings can be explained by slight natural variations in initial muscle glycogen content among sample batches and the subsequent lactic acid accumulation.

### 3.2. Effect of HE on Color of Raw Rabbit Meat

**Figure 2:**
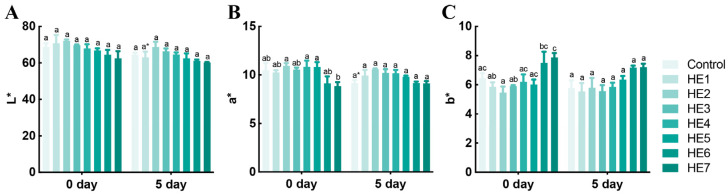
Changes in the color of raw rabbit meat treated by different concentrations of HE treatment during refrigerated storage. (**A**) L* value, (**B**) a* value, (**C**) b* value (*N* = 3 for each group). Data are expressed as mean ± SE. Different lowercase letters (a, b, c) indicate significant differences within the same storage time (*p* < 0.05) among groups. * Values within the same treatment indicate significant differences (*p* < 0.05).

The color was then evaluated in the raw rabbit meat. The results showed that the L* value of the raw rabbit meat was only significantly decreased by the HE1 group during the storage period (Figure 2A). The changes in the a* value of raw rabbit meat during refrigeration are shown in Figure 2B. All a* values ranged from 8.04 to 11.93. For the control group, the a* value of raw rabbit meat was significantly decreased on day 5, when compared with that found at day 0. On day 0, the raw rabbit meat treated with HE7 had a lower a* value than the HE2, HE4 and HE5 groups, and no change was noticed on day 5. For the b* value, there was no change in storage period, which ranged between 4.46 and 7.46 during the storage period (Figure 2C). However, the changes in b* values of raw rabbit meat were significantly affected by HE treatment on day 0; the b* values of raw rabbit meat treated with HE7 increased by 34.1% and 44.0%, respectively, compared with those in the HE1 and HE2 groups (Figure 2C).

### 3.3. Effect of HE on Lipid Oxidation in Raw Rabbit Meat

**Figure 3:**
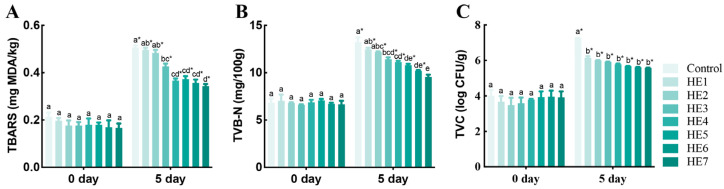
Changes in the quality preservation and lipid oxidation profiles of raw rabbit by different concentrations of HE treatment during refrigerated storage. (**A**) TBARS content, (**B**) TVB-N content, (**C**) TVC content (*N* = 3 for each group). Data are expressed as mean ± SE. Different lowercase letters (a–e) indicate significant differences within the same storage time (*p* < 0.05) among groups. * Values within the same treatment indicate significant differences (*p* < 0.05).

TBARS reflects the content of MDA produced during secondary lipid oxidation in rabbit meat and is one of the indicators used to evaluate the degree of lipid oxidation [23]. Thus, the role of HE in raw rabbit meat lipid oxidation was investigated. Results showed that HE treatment did not change the TBARS values compared to the control group on day 0 (Figure 3A). However, all the groups exhibited significantly elevated TBARS with prolonged storage time. In addition, the HE treatment groups (HE3 to HE7) had lower TBARS values on day 5, as compared with the control group (Figure 3A). These results indicated that the raw rabbit meat treated with HE treatment could delay lipid oxidation in raw rabbit meat.

### 3.4. Effect of HE on Antimicrobial Activity in Raw Rabbit Meat

The TVB-N value reflects the content of alkaline nitrogenous compounds produced by the spoilage and decomposition of proteins in rabbit meat and is used to assess its freshness [24]. Changes in TVB-N levels added without and with HE at different concentrations during 5 days of refrigerated storage are shown in Figure 3B. The results showed that TVB-N levels in rabbit meat treated with varying HE concentrations remained comparable to controls on day 0 of refrigerated storage. However, at day 5, the TVB-N values were significantly increased with prolonged storage time. Moreover, the control group showed the highest TVB-N value of 13.24 mg/100 g, while the HE treatment groups exhibited dose-dependent suppression of TVB-N accumulation at day 5, with values ranging from 9.55 to 12.47 mg/100 g. Furthermore, the HE7 group showed the strongest inhibition, achieving a 27.9% reduction compared with the control group. These data suggest that HE could reduce spoilage metabolites from protein degradation, consistent with its active compound-mediated antioxidant activity.

Next, TVC levels were investigated in raw rabbit meat with HE at different concentrations during 5 days of refrigerated storage. On day 0 the TVC of all groups ranged from 3.49 to 4.02 log CFU/g (Figure 3C), indicating the meat was clean and of high quality. When storage time was increased to day 5, the TVC levels were significantly higher than the initial levels. On day 5, the TVC value of the control group reached 7.27 log CFU/g and exceeded the limit (6–7 log CFU/g) for the meat, indicating that the meat had completely spoiled. However, compared with the control group, the HE treatment groups had lower TVC levels. These data suggest that HE treatment could inhibit the growth of bacteria damaging rabbit meat, and a higher concentration showed the enhanced antimicrobial activity more effectively.

### 3.5. Effect of HE on the Protein Oxidation of Raw Rabbit Meat

**Figure 4:**
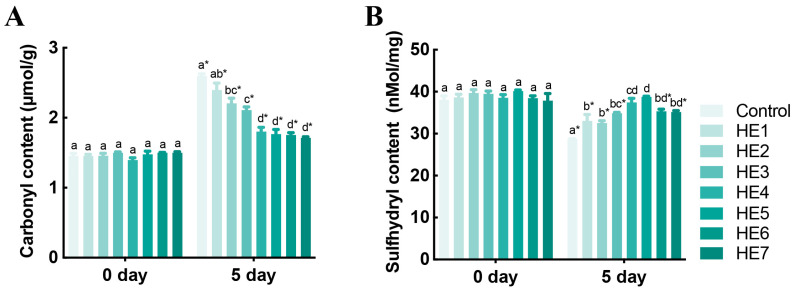
Changes in the protein oxidation profiles of raw rabbit by different concentrations of HE treatment during refrigerated storage. (**A**) Carbonyl content, (**B**) sulfhydryl content (*N* = 3 for each group). Data are expressed as mean ± SE. Different lowercase letters (a–d) indicate significant differences within the same storage time (*p* < 0.05) among groups. * Values within the same treatment indicate significant differences (*p* < 0.05).

The level of protein carbonyl content indicates the extent of protein oxidative damage and serves as a primary indicator for assessing such damage [25]. The change in total carbonyl content of rabbit meat during refrigerated storage is shown in Figure 4A. The carbonyl content was similar for all groups at day 0. As the storage time to day 5, the carbonyl contents were significantly increased compared with those in the initial levels. This result shows that the level of carbonyl content increased with extended refrigeration periods, indicating higher levels of protein oxidation. However, compared with the control group, HE treatment groups had a lower carbonyl content at day 5. Among them, the lowest carbonyl content was observed in HE7, whereas the highest carbonyl content was determined in the control group. In addition, no difference was determined between the HE1 and HE2 groups, nor between the HE4, HE5, HE6 and HE7 groups. These results suggested that HE treatment stabilizes MPs and inhibits protein oxidation in a dose-dependent manner.

The content of sulfhydryl was then investigated, and the results are shown in Figure 4B. On day 0, no differences in sulfhydryl contents were obtained for all groups, which ranged between 37.86 and 40.15 nmol/mg. The data indicated the HE treatment did not change sulfhydryl contents in the initial stage. However, with increasing storage time to day 5, all the groups, except HE4 and HE5, showed a reduction in sulfhydryl contents compared to day 0. On day 5, the highest protein sulfhydryl content was determined in the HE4 and HE5 groups, whereas the lowest was determined in the control groups. In addition, HE1 and HE2 groups had lower sulfhydryl content, compared to the HE4 and HE5 groups. The result indicated that adding 4% or 5% HE could have a positive effect of inhibiting protein oxidation.

### 3.6. Effect of HE on the Gel Water Retention of Rabbit MP

**Figure 5:**
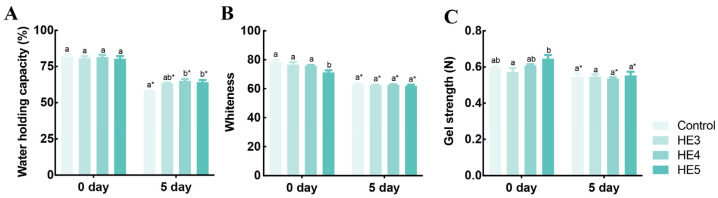
The effect of HE on the quality characteristics of rabbit meat. (**A**) Water-holding capacity, (**B**) whiteness, (**C**) gel strength (*N* = 3 for each group). Data are expressed as mean ± SE. Different lowercase letters (a,b) indicate significant differences within the same storage time (*p* < 0.05) among groups. * Values within the same treatment indicate significant differences (*p* < 0.05).

The water retention is one of the important qualities of protein gels and is closely related to the textural properties and water retention of meat products [26]. Figure 5A shows that the HE treatment and control groups showed a similar tendency of the WHC of rabbit meat protein gels on day 0. After 5 days of storage, all groups showed a decline in WHC levels compared to day 0. Moreover, compared with the control group, HE treatment groups had the higher WHC levels at day 5. Among them, the HE4 and HE5 groups had the highest WHC levels.

Whiteness is an important quality attribute of protein gels and has a direct impact on the color of rabbit meat products [27]. The results showed that the whiteness of the rabbit MP gels was significantly decreased during the storage period (Figure 5B). On day 0, the whiteness value of the rabbit MP gels in the control group was 63.03. After the addition of 5% HE, the protein gel whiteness significantly decreased (Figure 5B). However, the level of whiteness was similar for all the groups at day 5 (Figure 5B).

Gel-forming properties of proteins hold significant importance in the field of meat product processing and are strongly associated with the textural characteristics of meat products [28]. As shown in Figure 5C, with increasing storage time to day 5, all the groups, except HE3, showed a reduction in the gel strength of rabbit MP gel compared to day 0. In addition, during days 0 and 5 of the refrigeration storage processes, HE treatment did not change the gel strength of rabbit MP gel compared with the control, with values ranging from 55.06 to 69.34 g and 52.01 to 60.16 g on days 0 and 5, respectively.

### 3.7. Effect of HE on the Solubility of Rabbit MP

**Figure 6:**
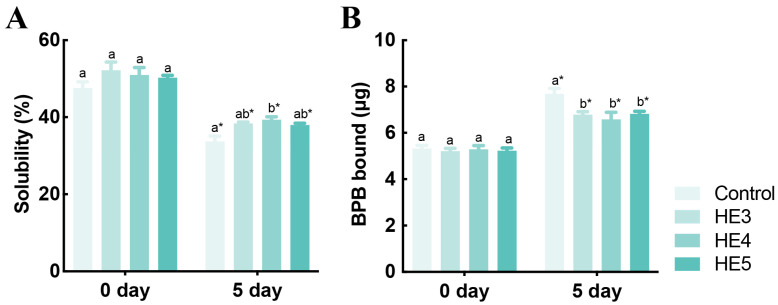
Changes in the muscle MP profiles of raw rabbit by different concentrations of HE treatment during refrigerated storage. (**A**) Solubility, (**B**) surface hydrophobicity (*N* = 3 for each group). Data are expressed as mean ± SE. Different lowercase letters (a,b) indicate significant differences within the same storage time (*p* < 0.05) among groups. * Values within the same treatment indicate significant differences (*p* < 0.05).

Protein solubility greatly influences protein function. As shown in Figure 6A, on day 0, no significant differences in protein solubility were observed among the groups, with values ranging from 47% to 50%. However, when the storage time increased to day 5, all groups exhibited a decrease in protein solubility levels compared to day 0. On day 5, the highest protein solubility levels were determined in the HE4 group, whereas the lowest was determined in the control group. These data suggest that HE treatment could prevent the generation of protein conglomerates.

### 3.8. Effect of HE on the Hydrophobicity of Rabbit MP

Next, the surface hydrophobicity of rabbit MP was analyzed. Due to its specific affinity for exposed hydrophobic domains, BPB binding capacity serves as a reliable index for evaluating protein surface hydrophobicity [29]. As shown in Figure 6B, compared with day 0, protein hydrophobicity increased significantly by day 5. In addition, HE treatment did not change the protein surface hydrophobicity of the raw rabbit meat compared with the control, with values ranging from 4.97 to 5.56 on day 0. However, when storage time was increased to day 5, HE treatment groups had lower surface hydrophobicity values, as compared with the control group.

## 4. Discussion

The study investigated the effects of HE on the oxidative stability, storage quality, and MP functionality of raw rabbit meat during a 5-day storage period at 4 °C. Firstly, the 4% HE treatment showed stronger effective inhibition of the rates of lipid (TBARS) and protein oxidation (carbonyl and sulfhydryl contents) in raw rabbit meat during refrigerated storage than the control group. In addition, HE treatment significantly inhibited the microbial growth in raw rabbit meat during refrigerated storage. Furthermore, the 4% HE treatment exhibited the highest WHC and solubility, and the lowest surface hydrophobicity in raw rabbit meat during refrigerated storage.

Color is a critical indicator for assessing meat quality; meat with a bright, attractive appearance is more acceptable to consumers [30]. Previous studies found that plant extracts can directly impart their inherent colors to the meat matrix [31,32]. Herein, on day 0, the raw rabbit meat treated with the highest concentration (HE7) exhibited a lower a* value than the HE2, HE4, and HE5 groups but a higher b* value than those of the HE1 and HE2 groups. These findings are attributed to the excessive deposition of intrinsic color of the HE, such as flavonoids and phenolic acids, which stained the meat surface and impacted the instrumental assessment of rabbit meat color. However, the effects of HE treatment on color acceptability and purchase potential of rabbit meat need further study to be explored.

The oxidative destruction of lipids is a major cause of quality deterioration in raw meat during refrigeration, resulting in off-flavors and the development of secondary oxidation products such as MDA, a key secondary peroxidation product evaluated as TBARS [33]. Rabbit meat is particularly susceptible to these reactions due to its high proportion of PUFAs. The current study found that as the storage period increased, all raw rabbit meat exhibited significantly greater TBARS levels, indicating continued oxidation during refrigerated storage. Similar findings were also reported in [34,35]. In addition, we showed that the HE treatment (HE3–HE7) significantly suppressed lipid oxidation on day 5, achieving an approximate 26–32% reduction in TBARS accumulation compared to the control group. Abdalrahman et al. reported that the rabbit meat treated with 0.2% plant extract mixture exhibited the lowest TBARS values and a 50% prolongation of shelf life under cold storage [36]. The study by Babiano et al. indicated that applying encapsulated purple sweet potato extract at 2500 and 5000 ppm to beef burgers resulted in approximately 28% and 44% reductions in TBARS, respectively, after 5 days of storage at 4 °C [37]. Previous studies reported that honeysuckle contains bioactive compounds such as polysaccharides, flavonoids, volatile oils, organic acids, and iridoid glycosides, which have antioxidant and anti-inflammatory activities [38]. These phytochemicals intervene in the lipid oxidation cascade primarily by acting as hydrogen donors to stabilize lipid peroxyl radicals and by chelating pro-oxidative transition metals naturally present in the heme pigments of the meat [39]. Our previous study demonstrated that honeysuckle extracted with 65% ethanol shows excellent antioxidant activity (DPPH and ABTS) and the optimal content of active compounds (TPC, TFC and CGA). Furthermore, we observed that 4% HE effectively delayed lipid oxidation, indicating a practical threshold for the extract at which a sufficient local concentration of phenolic antioxidants is achieved at the meat surface to intercept ROS before they propagate through the lipid bilayer. However, this should be further explored.

Lipid oxidation and protein degradation are recognized as prominent indicators of quality deterioration in meat, with acceptable spoilage limits defined at 0.5 mg MDA/kg for lipid oxidation and 15 mg/100 g for protein degradation [16], respectively, which should not be exceeded. The current study found that the level of TBARS and TVB-N were decreased by HE treatment, which means HE might prevent protein degradation by inhibiting lipid oxidation and reducing the generation of secondary lipid oxidation products, which inhibits these compounds from interacting with proteins. Furthermore, microbial proliferation is also a key parameter for evaluating meat freshness, with TVC values higher than 7 log CFU/g considered unacceptable spoilage [40]. The current study found that the control group reached the critical spoilage threshold on day 5, with TVC levels (7.27 log CFU/g) surpassing the acceptable limits. In contrast, the HE treatment groups remained below the limit on day 5 (5.59 to 6.39 log CFU/g). The antibacterial activity of bioactive compounds, particularly phenolics, is primarily mediated via ROS, cytoplasmic membrane disruption, and bacterial DNA damage [41]. Mahsa Hashemi et al. reported that the application of sodium alginate films containing 1% thymol to beef mortadella sausage showed stronger antimicrobial effects on TVC for 40 days at 4 °C [42]. Thus, the inhibited microbial growth in HE treatment rabbit meat might be due to the antimicrobial and anti-spoilage activities of the bioactive compounds in HE. Moreover, this decreasing trend was positively correlated with the TVB-N results (Figure 3B), further validating the dual inhibitory effects of HE treatment on microbial proliferation and protein degradation. These data suggest that HE could delay the quality deterioration and prolong the acceptable storage period of raw rabbit meat during refrigeration by inhibiting lipid oxidation, reducing spoilage metabolites from protein degradation and preventing the growth of bacteria. However, the exact mechanisms by which HE maintains the refrigerated quality of rabbit meat need further study to be elucidated.

In addition to lipid oxidation, protein oxidation, typically manifested by increased carbonyls and decreased sulfhydryl groups, is a primary driver of meat quality deterioration. Sulfhydryl groups, primarily derived from cysteine residues, are highly susceptible to ROS attack, readily forming disulfide cross-links that induce protein aggregation and functional impairment [43]. Previous studies found a reduced trend in sulfhydryl group content after refrigerated storage of rabbit meat [44,45]. Herein, we found that, with the exception of the HE4 and HE5 groups, the sulfhydryl content was significantly reduced in all groups after storage, as compared with their initial levels. The explanation for this result might be that as the storage time increases, the sulfhydryl groups are oxidized and transformed into disulfides [46]. In addition, the current study showed that the sulfhydryl content first increased in the HE4 and HE5 groups, followed by a decline in the HE6 and HE7 groups. Previous studies found that natural antioxidants obtained from plants, such as gallic acid and rutin, could reduce the sulfhydryl content of meat protein from pork [47,48]. The sulfhydryl loss may be mediated by the induction of the electrophilic quinone carbonyls generated by phenolic oxidation and nucleophilic cysteine sulfhydryl groups [49]. However, low concentrations of phenolics may play a positive role, as non-disulfide covalent connections occurred between quinones and nucleophile sulfhydryl groups. Thus, the content of sulfhydryl was decreased, which may also be caused by the covalent interactions between phenolics and sulfhydryl groups [48]. Similar results were confirmed in previous studies [50]. Further investigations revealed that HE treatment exhibited lower carbonyl content in raw rabbit meat during refrigerated storage. Notably, a strong “lipid–protein co-oxidation” cascade exists in the complex meat matrix. Primary lipid radicals and secondary reactive carbonyl species generated from lipid oxidation can migrate to the aqueous phase and directly attack basic amino acid residues, such as lysine, arginine, and histidine to form protein carbonyls [51]. The current study found that the reduction in carbonyl accumulation was directly associated with the decrease in TBARS, indicating that HE effectively intercepts lipid radicals, thereby cutting off the supply of reactive carbonyl species and preventing the lipid–protein co-oxidation cycle. Interestingly, the HE treatments exhibited a non-monotonic, dose-dependent effect on sulfhydryl preservation. The sulfhydryl content was optimally maintained at 4% and 5% HE, but sharply declined at 6% and 7% HE, despite these higher doses exhibiting the lowest carbonyl contents. This phenomenon may be caused by phenolic–protein covalent interactions. At higher concentrations, ortho-diphenols in the extract can auto-oxidize into highly electrophilic quinones. These quinones readily undergo Michael addition with nucleophilic sulfhydryl groups, forming non-disulfide covalent bonds [52]. This process chemically hides the free thiol groups, imitating oxidative loss, resulting in excessive protein cross-linking. Thus, HE, especially at 4% and 5% mg/kg, showed the optimal critical concentration that maximizes radical scavenging and prevents lipid–protein co-oxidation, without triggering detrimental quinone-induced structural interference. However, a more comprehensive comparison between HE and synthetic antioxidants, including antioxidant properties, needs further study to be explored.

The structural conformation of MP has a fundamental impact on their functional characteristics, particularly solubility and WHC. During refrigerated storage, the natural tertiary structures of myosin and actin are highly susceptible to attack by ROS, which induces spatial unfolding of the native myosin α-helices. This unfolding exposes buried non-polar aliphatic and aromatic amino acid residues to the aqueous environment, and these hydrophobic patches drive protein aggregation into insoluble conglomerates, ultimately altering overall solubility [51]. In the current study, compared to day 0, the surface hydrophobicity was increased, and the protein solubility was decreased in all groups during the 5 days of refrigerated storage, indicating the occurrence of oxidative denaturation. Zhang et al. found that the BPB binding capacity was increased and MP solubility was decreased in pork samples during refrigerated storage [53]. Similar findings were also reported in [54]. Moreover, some studies have pointed out that active compounds, such as several simple phenols and flavonoids, could interact with MP through hydrogen bonds to reduce surface hydrophobicity to improve the structural and functional properties of MP [55]. Cao et al. showed that (−)-Epigallocatechin-3-gallate (EGCG) decreased the surface hydrophobicity of porcine MP [56]. The study by Sun et al. indicated that the extract of *Flos sophorae immaturus* (FSI) and its main active ingredient, rutin (52% of the FSI extract), might attenuate the influence of hydrophobic groups in Mb through non-covalent interactions while concurrently enhancing overall polarity via their inherent hydroxyl moieties [30]. The current study showed that HE treatment significantly decreased the binding rate of BPB and increased the protein solubility compared to the control group, indicating HE effectively intercepted the occurrence of oxidative denaturation pathway. Thus, this phenomenon may be explained by active compounds (TPC, TFC and CGA) in HE, which accumulate at the protein–water interface, where they directly quench hydroxyl and peroxyl radicals, preventing them from initiating the radical chain reactions responsible for protein unfolding. By safeguarding sensitive hydrophobic segments against radical attack, HE might preserve the native, highly soluble conformation of rabbit MPs, which is concomitant with the suppression of carbonyl formation, ultimately ensuring the attainment of an unaggregated protein state during the heat-induced gelation process. Importantly, the preservation of WHC is closely correlated with this oxidative stabilization. The ability of raw meat to hold moisture is primarily determined by changing the structure and spatial arrangement of MP [57]. The current study found that the levels of WHC were significantly increased in raw rabbit meat receiving 4% or 5% mg/kg HE treatment compared to the control group. The increased WHC level was consistent with the protein solubility results (Figure 6A) in this study, indicating that HE treatment inhibits disulfide cross-linking and quinone–thiol adduction, preserving electrostatic repulsion and the native structure of thick and thin filaments. This prevents shrinkage of the myofibrillar lattice, preserving capillary forces that hold water within the muscle matrix.

Interestingly, HE treatment effectively improved WHC, although the value of gel strength was not changed. This apparent difference might be partially attributable to the differences in physicochemical characteristics of water retention and thermal gelation. WHC relies primarily on maintaining the native spacing of the myofibrillar lattice to entrap water by capillary forces during refrigeration. Conversely, the development of a strong, elastic three-dimensional gel network during heat induction strongly depends on the formation of protein–protein cross-links, particularly covalent disulfide bonds [58]. At low doses (6–30 μM/g protein), chlorogenic acid and gallic acid promote disulfide and other covalent cross-links in oxidized pork MP, improving gelation. However, high doses (150 μM/g) favor thiol–quinone adduct formation over beneficial cross-links, which markedly reduces gel quality [59,60]. Similar findings were also reported in [61]. HE effectively scavenges radicals and inhibits the oxidation of sulfhydryl groups into disulfides, which preserves quality during refrigeration but limits the gel-reinforcing bonds needed for thermal processing, resulting in improved water retention but no change in gel strength. Overall, HE, especially at 4% and 5% mg/kg, serves as a highly potent natural preservative that reduces oxidative denaturation and maintains the functional integrity, solubility, and WHC of rabbit meat during refrigerated storage. However, the exact mechanism for the interaction between specific phenolic compounds in HE and individual amino acid residues on MP during refrigerated storage needs further study to be elucidated.

## 5. Conclusions

In summary, this study revealed that HE treatment significantly suppresses both lipid and protein oxidation while inhibiting microbial proliferation in raw rabbit meat over a 5-day refrigerated storage period. By mitigating oxidative attack, HE effectively preserves the structural and functional integrity of MP. Consequently, HE shows strong potential as a natural preservative to maintain the storage quality of raw meat. However, considering the limitations of this 5-day observational study, future research incorporating formal sensory evaluation, microbial community analysis, and industrial validation is required to fully confirm its commercial viability and long-term preservation efficacy.

## Data Availability

The original contributions presented in this study are included in the article. Further inquiries can be directed to the corresponding author.
